# Utilization of a novel siRNA platform technology to target APOE in the brain for the treatment of Alzheimer’s Disease

**DOI:** 10.1002/alz70859_105053

**Published:** 2025-12-26

**Authors:** Maxine R. Nelson, Marina Kovaliov, Priyanka Singh, Akila Ram, Clara Tourino, Li Li, Kaylin H. Hwang, Priya Pulya, Alex Embusch, Shilpi Mahajan, Lisa J. Scherer, Abhinav Kumar, Donald C. Button, Robert Duff, Charles R. Allerson, Dee Datta, Anindya Bhattacharya, Craig Blanchette

**Affiliations:** ^1^ Switch Therapeutics, South San Francisco, CA USA

## Abstract

**Background:**

The greatest genetic risk factor for late‐onset Alzheimer’s disease (AD) is Apolipoprotein E (APOE)*ε4. Preclinical studies have demonstrated that genetic ablation or reduction of APOE4 mRNA in amyloid and tauopathy mouse models have protective effects and/or reduce pathology. Targeting central APOE, while sparing systemic APOE and cholesterol metabolism, is the holy grail for a precision medicine approach in APOE*ε4 carriers with MCI/AD. Here, we present data for the first time, demonstrating our novel, highly potent, conditionally activating siRNA (CASi) targeting APOE mRNA in the brain while sparing peripheral APOE.

**Method:**

A library of CASi and parental siRNA molecules targeting APOE was designed and tested in iPSC astrocytes. The in vivo activity of the most potent compounds was evaluated in human APOE4 transgenic (hE4) mice over a dose concentration range. Activity was evaluated in both brain regions and the liver for lead selection. The lead molecule was further evaluated in nonhuman primate (NHP) studies, administered intrathecally at 112mg, 168mg, or 224mg doses. Knockdown of CSF, brain and liver tissue APOE was assessed at multiple time points.

**Result:**

The potency of the lead CASi molecule was <20pM in iPSC astrocytes. A 10nmol dose yielded 60‐85% reduction of APOE mRNA in hippocampal tissue at 2ng/mg CASi exposure. The molecule was well‐tolerated in mice. No knockdown was observed in the liver, despite CASi exposure, and peripheral cholesterol levels were unchanged. The dose‐response study demonstrated a tight PK/PD relationship with a significant (P<0.0001) negative correlation between CASi and APOE mRNA levels in hE4 mice. NHP studies demonstrated 50‐75% APOE protein knockdown in the CSF 15 days post dosing, which was sustained until day 70. At 30 days post‐dose, the entorhinal cortex and hippocampus showed a 50‐75% knockdown. There was no reduction of APOE protein levels in liver or plasma across the study.

**Conclusion:**

We identified a potent CASi against APOE with no unwanted effects on liver APOE levels or peripheral cholesterol. The molecule holds promise as a future therapeutic for ε4 carriers suffering from MCI/AD, many of whom are contraindicated for the currently approved immunotherapies.

